# Identification of a 57S translation complex containing closed-loop factors and the 60S ribosome subunit

**DOI:** 10.1038/s41598-018-29832-6

**Published:** 2018-07-31

**Authors:** Clyde L. Denis, Thomas M. Laue, Xin Wang

**Affiliations:** Department of Molecular, Cellular, and Biomedical Sciences, University of New Hampshire, Durham, NH 03824 USA

## Abstract

In eukaryotic translation the 60S ribosome subunit has not been proposed to interact with mRNA or closed-loop factors eIF4E, eIF4G, and PAB1. Using analytical ultracentrifugation with fluorescent detection system, we have identified a 57S translation complex that contains the 60S ribosome, mRNA, and the closed-loop factors. Previously published data by others also indicate the presence of a 50S-60S translation complex containing these same components. We have found that the abundance of this complex increased upon translational cessation, implying formation after ribosomal dissociation. Stoichiometric analyses of the abundances of the closed-loop components in the 57S complex indicate this complex is most similar to polysomal and monosomal translation complexes at the end of translation rather than at the beginning or middle of translation. In contrast, a 39S complex containing the 40S ribosome bound to mRNA and closed-loop factors was also identified with stoichiometries most similar to polysomal complexes engaged in translation, suggesting that the 39S complex is the previously studied 48S translation initiation complex. These results indicate that the 60S ribosome can associate with the closed-loop mRNA structure and plays a previously undetected role in the translation process.

## Introduction

The process of eukaryotic protein translation occurs through multiple steps: initiation, elongation, termination, and recycling of ribosomes^1–5^. In the current model for protein synthesis in eukaryotic organisms, such as the yeast *Saccharomyces cerevisiae*, the mRNA initially forms a closed-loop structure in which eIF4E, the mRNA cap binding protein, binds eIF4G, which in turn binds the poly(A)-binding protein (PAB1) that is bound to the poly(A) tail of mRNA^6^. This structure would link the 5′ end of the mRNA to its 3′ end. The resultant complex subsequently interacts with the 43S complex (40S small ribosomal subunit, translation initiation factors eIF2, −3, −5, 1A, and −1, and the charged methionine tRNA) to form the 48S complex^7^. This 48S complex then scans for the initiation codon and, as aided by other initiation factors, binds the 60S large ribosomal subunit to form a 77S monosomal translating complex consisting of the 80S ribosome bound to the mRNA^8^. Following elongation and protein formation, translation termination involves eRF1 recognition of the stop codon which, in conjunction with other proteins such as eRF3 and DBP5, ends protein synthesis^9–11^. The 80S complex is then recycled using such factors as RLI1, HBS1, DOM34, and eEF3^12–15^.

The identification of translation complexes and their components has been determined in part by *in vitro* reconstitution experiments, which by their nature may not be indicative of the complexes and the components of the *in vivo* situation^16–18^. Techniques have also been developed to isolate the 43S, 48S, and other translational complexes from crude extracts by sucrose gradient analyses and thereby characterize the complexes as they would exist *in vivo*^19–24^. There are several limitations that inhere, however, to sucrose gradient analyses in the identification of protein complexes. The nature of sucrose gradient analyses is such that a sampling across the centrifuge tube for Western analysis is usually limited to about 15 to 20 samples, resulting in loss of precision as to the identification and sizing of complexes. Overlapping complexes may not be identified, e.g., sucrose gradient analysis cannot discern between the free 80S ribosome and the 77S monosomal translating complex^8,25^.

Analytical ultracentrifugation analysis (AUC) allows the rapid determination of the size of protein/RNA complexes^26,27^. To maximize the advantages of AUC, we have used an AU instrument with a fluorescence detection system (AU-FDS)^28,29^. In this case, proteins or individual mRNA species that are fluorescently tagged with GFP^30^ can be identified uniquely^8,25,31–34^. A single AU-FDS experiment, without resorting to Western analysis, can determine unambiguously and precisely all of the protein/RNA complexes containing a particular GFP-tagged entity. As compared to sucrose gradient analysis, which essentially only takes one snapshot across a centrifuge cell, AUC is both able to sample several hundred positions across the cell and to take up to 900 scans to identify protein complexes, making it at least an order of magnitude more precise. AUC analysis is typically conducted in physiological buffers without the presence of high concentrations of sucrose that may result in compounding effects (e.g., the false forcing of aggregation due to the medium’s high density) and at 20 °C, a temperature closer to physiological conditions than that used for sucrose gradient analysis (4 °C). Therefore, the application of AU-FDS to identifying novel complexes should provide significant improvements over current technologies and lead to new findings^8,25,31,32^.

We have used different affinity purification steps combined with AU-FDS to characterize translational complexes from the yeast *Saccharomyces cerevisiae*^8,25,31,33,34^. In the course of these studies, we have identified a 57S translation complex that has not previously been remarked upon. The 57S complex contains the 60S ribosome subunit bound to mRNA and the closed-loop factors, eIF4E, eIF4G, and PAB1. We have found that the abundance of this complex increases upon translational cessation and less so during re-initiation of translation, suggesting that this novel translation complex plays roles in ribosomal recycling and initiation. Current models of translation do not indicate that the 60S ribosome associates with mRNA in such a complex upon termination. For example, previous studies did not directly measure mRNA presence on the 60S subunits released upon dissociation, assuming polysomal decreases were consonant with mRNA dissociation^12^. A number of other previous studies show clear evidence, under the same conditions that we have used, of a 50S to 60S complex containing the same factors that we have found, confirming its existence by other techniques^35–39^. Previous studies on defining the complexome of eRF1 also identified eRF1-containing 57S and 39S complexes, but in these cases the abundance of closed-loop factors was extremely low and these complexes were hence different in nature that the complexes reported here^31^. The significance of these observations is that our understanding of protein translation remains incomplete and that ribosomal interactions previously not conceived are critical to a comprehensive understanding of the translational process.

## Results

### Detection of complexes containing PAB1 and eIF4E

**Figure 1:**
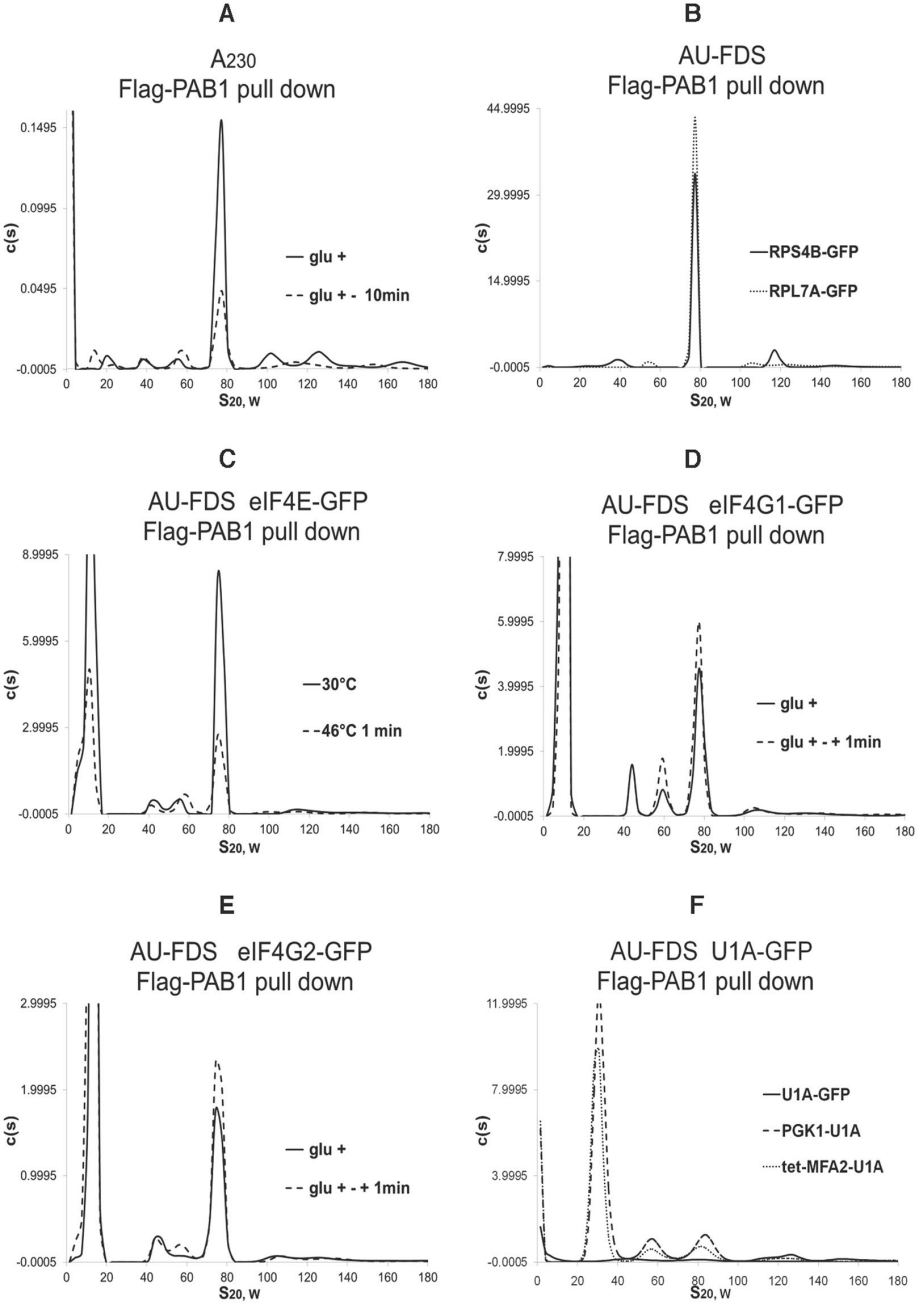
AUC identification of the 57S complex following Flag-PAB1 pull downs. AU-A_A230_ and AU-FDS analyses on Flag-PAB1 pull downs on cell extracts were done at rotor speeds of 15K rpm. Glucose growth conditions (glu +  or 30 °C) are a proxy for steady-state elongation whereas glucose deprivation conditions (glu+ − 10 min) or heat shock conditions (46 °C 1 min) result in translational repression^8,25,46^. Initiation conditions were determined by adding glucose back for 1 min to glucose deprived cultures (glc+ - +1 min). Data displayed in the same figure were conducted on the same centrifugation run. It should be noted that differences in the c(s) values (y-axis) between all experiments conducted on different days can not be judged to be significant due to several factors. Cycloheximide was added to all cells prior to cell lysis to prevent ribosomal run-off from mRNA during preparation. (**A**) AU-A_230_ analysis of Flag-PAB1 pull down showing glc +  versus glc+ - (**B**–**E**). AU-FDS analysis on Flag pull downs on strains carrying GFP tag proteins as indicated under the conditions described. (**F**) AU-FDS analysis on a Flag-PAB1 pull down in strains carrying *PGK1-U1A*, *tet-off-MFA2pG-U1A*, or no mRNA co-expressed in cells expressing *U1A-GFP*.

Translation complexes containing PAB1 were purified by a one-step purification of Flag-PAB1 expressed in yeast growing exponentially on glucose-containing medium^8^. Initially, AU-A_230_ analysis was used to detect all complexes containing Flag-PAB1. As shown in Fig. 1A, in addition to the 77S monosomal translating complex and polysomes (complexes migrating greater than 90S)^8,25^, three smaller complexes were identified: 20S, 39S, and 57S (for 53 analyses the 39S complex was 38.9S ± 0.30, Standard Error of the Mean, and the 57S complex was 56.6S ± 0.36). Material migrating at about 8S was not considered further, as it is indicative of proteins that are about 120 KDa if spherical. The identity of components within these three new complexes was determined by subjecting Flag-PAB1 purified material to AU-FDS analysis using strains containing different GFP tagged translation factors^8,25,31,34^. Small ribosomal subunit proteins RPS4B, RPS4A, and RPS30B were found to co-migrate with the 39S complex but to be absent from the 20S and 57S complexes (Fig. 1B; Suppl. Figure 1A,B,D). Similarly, large subunit proteins RPL6B, RPL7A, RPL7B, RPL12A, and RPL14B were found to migrate at 57S but to be absent from the 20S and 39S complexes (Fig. 1B; Suppl. Figure 1C–E). In contrast, eIF4E, eIF4G1, and eIF4G2 were found to be present in 39S and 57S complexes (Fig. 1C–E, respectively) but not to be present in the 20S complex. These results establish that the 39S and 57S complexes contain the closed-loop factors, eIF4E, eIF4G, and PAB1, apparently associated with an mRNA through eIF4E binding to the mRNA cap and PAB1 binding to the polyadenylated tail. The 39S complex contained the 40S ribosome and closed-loop factors, making it most similar to the 48S complex. The 57S complex contained the 60S ribosome and closed-loop factors, a complex that had not previously been known to exist. The 57S complex is not the same as the 60S complex formed upon ribosome stalling^40,41^, as this latter complex lacks the closed-loop factors. The 20S complex that was devoid of ribosomes was not studied further.

The presence of mRNA in these complexes was determined by co-expressing U1A-GFP in strains carrying mRNA tagged in their 3′ UTR sequences with binding sites for the U1A protein: *PGK1pG-U1A* and *tet off-MFA2-U1A*^8,42,43^. These two mRNA, following Flag-PAB1 pull downs and AU-FDS analysis, were present in 39S and 57S complexes in addition to the 77S translating monosomal and polysomal complexes (Fig. 1F). Strains expressing only the U1A-GFP protein did not display appreciable levels of the 39S and 57S complexes following AU-FDS analysis of Flag-PAB1 pull down material.

Previous analyses with other translation initiation factors (eIF2α, eIF3b, eIF4A, and eIF5) indicated that they were not present in 77S, polysomal, or 39S and 57S complexes (see ref.^8^ Suppl. Figure 2A). Because it had been shown previously that the 48S complex containing the eIF3 complex is unstable^16,21,31^, we performed the Flag-PAB1 pull downs in the presence of formaldehyde that is known to stabilize the 48S complex and association of factors with mRNA bound to PAB1^21,31,34,44^. In this case, a small percentage of eIF3b was found in the 77S monosomal translating complex and much more significant amounts were present in a complex migrating around 39S (Suppl. Figure 2B)^31^. However, eIF3b did not migrate in a 57S complex. These results suggest that eIF3b can be present in the 39S complex, making this complex most similar to the 48S pre-initiation complex. Prior analysis of the size of the 48S complex by sucrose gradient analysis actually does tend to position it slightly under or at 40S and not at 48S (see, for instance, Fig. 3D in ref.^45^), consistent with our AUC analysis (which would be used to define the real S value).

**Figure 2:**
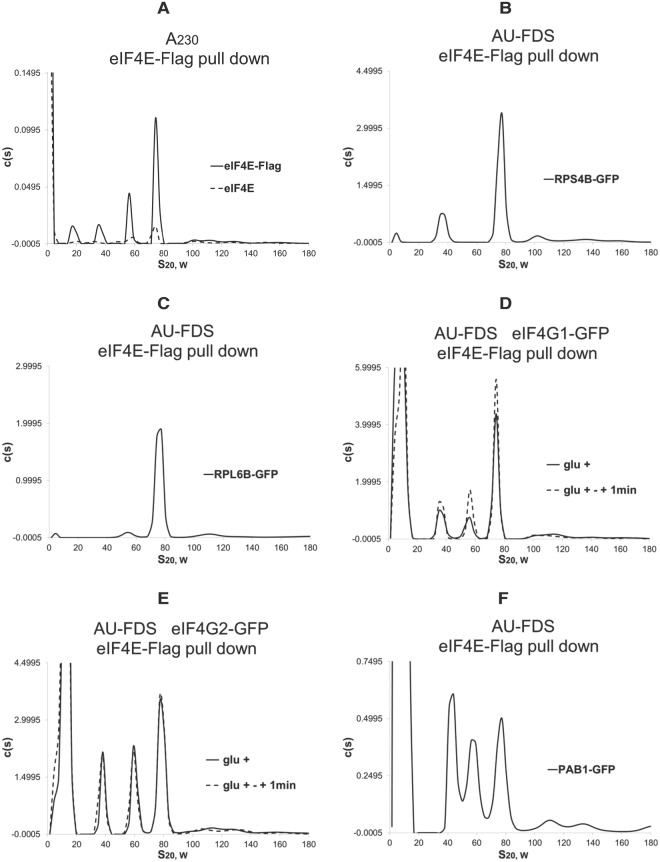
AUC analysis following eIF4E-Flag purifications. AUC analyses were conducted as described in Fig. 1 except eIF4E-Flag pull downs were conducted^25^. (**A**) AU-A_230_ analysis displaying eIF4E-Flag pull down as compared to an isogenic strain lacking eIF4E-Flag. (**B**–**F**) AU-FDS analyses were conducted on the GFP tag proteins as indicated under the conditions described.

Because eIF4E was found to be present in a 57S complex containing the 60S ribosome, we subsequently used eIF4E-Flag to analyze the 57S complex^25^. As shown in Fig. 2A, 57S, 39S, and 20S complexes were identified by AU-A_230_ analysis following an eIF4E-Flag pull down. Similarly, RPS4B was localized to the 39S complex (Fig. 2B), but not to the 57S and 20S complexes, and RPL6B was found in the 57S complex but not the 39S and 20S complexes (Fig. 2C). As expected, the 77S complex contained both RPS4B and RPL6B, as previously demonstrated^8,25^. Closed-loop factors eIF4G1, eIF4G2, and PAB1 were present in all the 39S and 57S complexes but not in the 20S complex (Fig. 2D–F, respectively). These are the same results obtained with Flag-PAB1 pull downs. It should be noted, however, that the c(s) values for eIF4G1, eIF4G2, and PAB1 in the 39S and 57S complexes relative to the values detected in the 77S monosomal translating complex following eIF4E-Flag pull downs were several-fold higher than the relative levels of the closed-loop factors in Flag-PAB1 complexes. These observations suggest a high level of abundance of closed-loop factors in the 57S and 39S complexes for those complexes that contain eIF4E (see below for a stoichiometric analysis).

### The 57S complex does not result from artifactual breakdown of translational complexes during cell lysis

**Figure 3:**
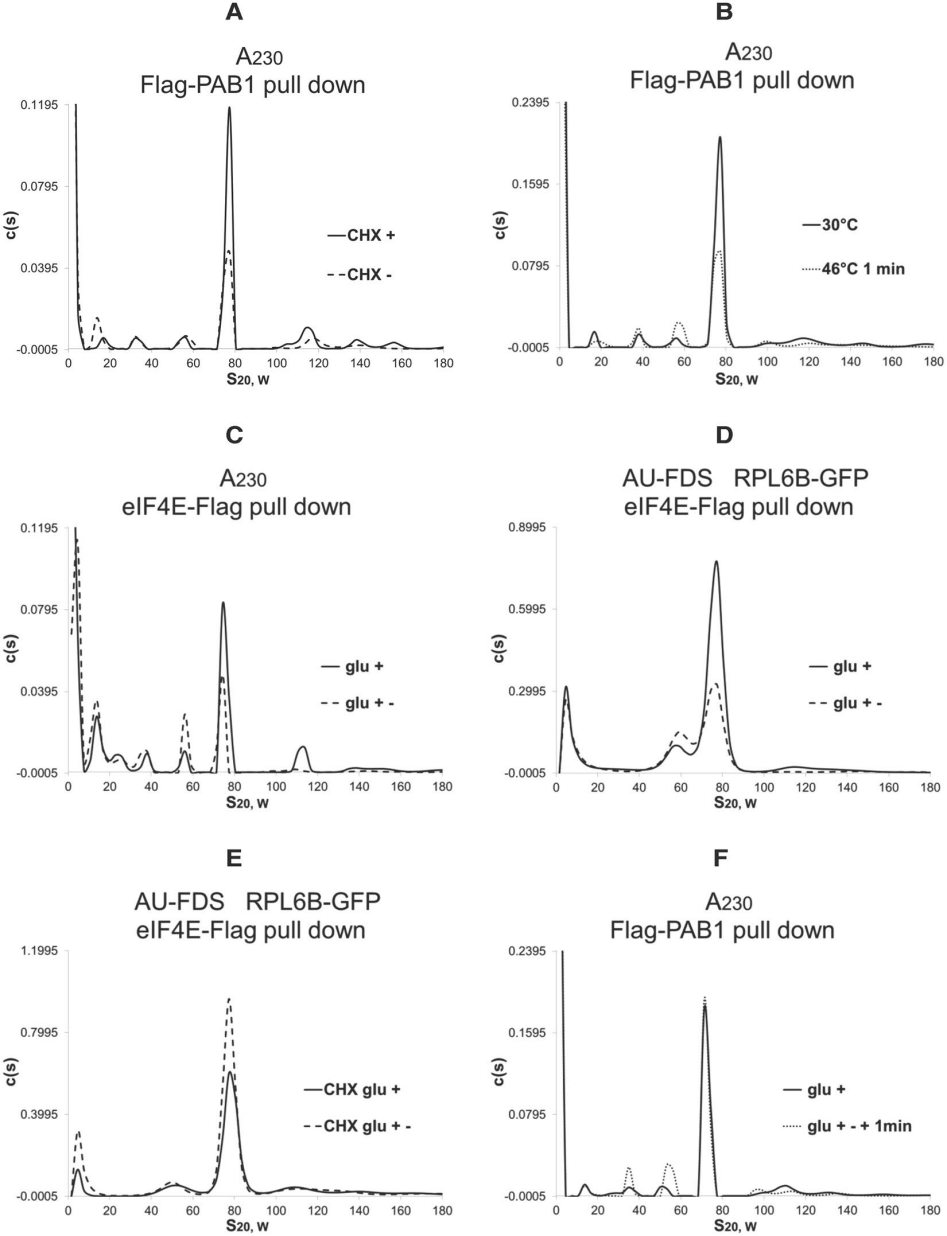
AUC analyses following Flag-PAB1 and eIF4E-Flag purifications. Experiments were conducted as described in Fig. 1. Cycloheximide experiments were conducted as described in the Materials and Methods. For panel A, cycloheximide was either added to cells prior to lysis (CHX+) or not (CHX−). For panel D, cycloheximide was added after the indicated treatment as normal as described in the Materials and Methods, whereas in panel 4E cycloheximide was added for 10 min prior to lysis (CHX glu+) or for 10 min before the shift to glucose depleted medium that was then followed by cell lysis (CHX glu+-).

One possibility for the existence of the 57S complex is that it is the result of breakdown of translational complexes during lysis of the cells and preparation of cell extracts. All of the above experiments (except the formaldehyde experiments) were conducted in the presence of cycloheximide that would have maintained the translating ribosomes on the mRNA after cell lysis^8,25,31^. To examine this further, we compared by AU-A_230_ analysis Flag-PAB1 pull downs of extracts from cells that had been treated with cycloheximide with extracts where cycloheximide had not been added. As shown in Fig. 3A, Flag-PAB1 pull downs detected in the cycloheximide treated cultures about twice as much 77S monosomal and polysomal translating complexes as compared to the non-cycloheximide treated cultures (consistent with previous observations indicating translation complex breakdown in the absence of cycloheximide)^8^. However, the 39S and 57S complexes did not increase in abundance in the absence of cycloheximide. Similar results were obtained with eIF4E-Flag pull downs (Suppl. Figure 2C). These observations indicate that these two complexes are not the result of degradation of translating complexes during post-*in vivo* manipulations.

### Translational termination specifically increases 57S complex abundance

The above data indicate the existence of a 57S complex containing the 60S ribosome, mRNA, and closed-loop factors. Previously published data has also identified 50S to 60S translation complexes that are distinct from the free 60S ribosome and which contain PAB1, mRNA, and closed-loop factors^35–39^. These complexes that are observed in these studies appeared to increase in abundance under conditions that increased translational termination in the cell (see Discussion for a complete analysis of these studies).

To investigate the functional role of the 57S complex, we analyzed its changes in abundance following translation termination and during translation initiation. Our reasoning was that if the 57S complex were important to translational processes, then different effects on translation would alter its abundance. As an internal control, we followed the 39S complex (most similar to the 48S complex). The abundance of the 39S complex should increase under conditions when new initiation was occurring, not necessarily increase as ribosomes run off the mRNA after termination, and its stoichiometric ratios of closed-loop factors should be similar to that of polysomal complexes to which it is a precursor. We first studied conditions that modeled increased translational repression and termination (a heat shock treatment by shifting yeast for 1 min from 30 °C to 46 °C)^8,46^. Growth at 46 °C for 10 min represses translation due to ribosomal run-off from the mRNA^8,46^ and growth at 1 min would be the beginning of this process. Under these conditions, we found that the 77S monosomal translating complex was reduced to 64% (see Table 1) of the levels found under normal elongation conditions and polysomal abundance decreased to 54% of normal, consistent with the commencement of translational cessation due to heat shock (Fig. 3B). Importantly, translation termination factor eRF1 was found to display a two-fold increase in association with the 77S complex under such conditions whereas the closed-loop factors eIF4E and eIF4G abundances remained relatively constant (Suppl. Table 1). These results are consistent with this 1 min shift to high temperature serving as a proxy for increased translation termination events.

**Table 1:** Relative levels of 39S (48S) and 57S complexes following translational shut-off or initiation as detected with AU-A_230_ analysis following Flag-PAB1 pull downs.

	Elongation	Termination	Termination	Initiation	
Complex	Glu +	30 °C to 46 °C 1 min	Glu + − 10 min	Glu + − 10 min +1 min	*cdc33-1*
39S (48S)	100%	140 ± 14 (20)	110 ± 15 (19)	150 ± 16 (23)	74 ± 18 (3)
57S	100%	220 ± 14 (21)	190 ± 26 (19)	300 ± 25 (23)	123 ± 11 (3)
77S monosomal translating complex	100%	64 ± 2.6 (28)	36 ± 1.7 (15)	94 ± 2.1 (37)	49 ± 4.5 (4)
polysomal	100%	54 ± 6.8 (23)	46 ± 4.9 (19)	62 ± 7.7 (32)	38 ± 6.7 (3)

The relative levels (in percentage) of the 39S (similar to the canonical 48S complex) and 57S complexes (± Standard Error of the Mean, S.E.M.) were compared to steady-state elongation conditions (Glu +) (set at 100) following Flag-PAB1 pull downs and AU-A_230_ analysis. The relative levels of the 77S monosomal translating complex and that of polysomes are given as comparisons and are taken from Wang *et al*. 2012 and 2016^8,25^. Translational repression was conducted either for 10 min by depleting glucose from the medium or by shifting glucose-grown cells to 46 °C for 1 min^25^. Reinitiation of translation was accomplished by adding glucose back to glucose depleted cultures for 1 min. The *cdc33-1* alelle was grown at 37 °C for one hr and compared to the isogenic parent strain grown under the same conditions^8^. The number of samples for each analysis is in parenthesis after the S.E.M. value.

Following this shift of yeast for 1 min to 46 °C, the 57S complex was found by AU-A_230_ analysis to increase in abundance 2.2-fold whereas the 39S complex increased 1.4-fold (Table 1). To examine another condition when translational run-off and ribosomal dissociation would be occurring, we subjected yeast to translational repression by growth for 10 min in medium lacking glucose^8,25,47^. Under such conditions the 77S monosomal translating complex decreases to 36% of that found under steady-state elongation conditions and the polysomal abundance drops to 46% of steady-state elongation conditions (Table 1)^25^. Consistent with these conditions representing increased termination events, eRF1 abundance under these conditions increased four-fold in the 77S monosomal translating complex, whereas, comparatively, eIF4E and eIF4G abundances remained relatively constant (Suppl. Table 1). Under these glucose depletion conditions, the 57S complex abundance increased by 1.9-fold whereas the 39S complex abundance did not change (Fig. 1A; Table 1). The data from these two sets of conditions that model translational termination are consistent with the 57S complex forming after 80S ribosome dissociation upon translation termination in which the mRNA and closed-loop factors stay attached to the 60S ribosome. The 39S complex, however, may only be partly forming as a result of translational shut-off. Similarly, with eIF4E-Flag purified material, following AU-A_230_ analysis, the 57S complex increased by 1.9-fold (±0.15 for seven samples) following glucose depletion while the 39S complex increased by 1.3-fold (±0.10 for seven samples) (Fig. 3C).

As an additional control for these experiments involving glucose depletion from the medium, we tested whether blocking translational run-off prior to removing glucose from the medium would affect 57S complex abundance. In this scenario, if the increase in 57S abundance were a result of translational run-off and 80S ribosomal dissociation into some complexes containing the 60S subunit still bound to mRNA and closed-loop factors, adding cycloheximide prior to depleting glucose from the medium should block the increase in 57S complex abundance. Using eIF4E-Flag pull downs in the presence of RPL6B-GFP to follow the 57S complex, we found that RPL6B abundance in this complex increased 1.8-fold after glucose was depleted from the medium (Fig. 3D) but that its abundance in the complex did not increase if cycloheximide were added prior to depletion of glucose from the medium (Fig. 3E). These results indicate that the 57S complex is specifically forming upon termination and translational run-off and verify that cycloheximide does prevent translational run-off^31^.

### The 39S complex abundance but not that of the 57S complex is reduced by blocking translation initiation

To determine if the 57S complex plays any role in initiation of translation, we used conditions where re-initiation of translation is modeled (adding glucose back for 1 min to glucose depleted cultures in which 77S monosomal translation complex abundance returns to 94% of that seen previously in glucose-grown cells under steady-state conditions) (Table 1)^25^. We found following AU-A_230_ analysis that the 57S complex abundance increased by 1.6-fold from the repressed conditions (3.0-fold from glucose conditions), and, as expected, the 39S complex increased in abundance by 1.4-fold (1.5-fold over glucose conditions) (Fig. 3F). Therefore, the 57S complex forms both upon translational cessation and when new initiation occurs.

To further analyze the 57S complex in terms of initiation, we blocked initiation using a mutation in eIF4E (*cdc33-1*) that binds the mRNA 5′ cap less well at the restrictive temperature^8,48^. This mutation inhibits translation initiation at a step prior to 48S complex formation^21^, apparently at the formation of the closed-loop structure^8,49^, and correspondingly reduces the monosomal translation complex abundance by 2.0-fold and that of the polysomal material by 2.6-fold (Table 1). In a *cdc33-1* background at the restrictive temperature, the 39S complex decreased in abundance by 1.5-fold, as expected from a 48S complex in which initiation is blocked. In contrast the 57S complex did not change in abundance (1.0-fold) (Table 1) (Suppl. Figure 2D). The fact that the 57S complex abundance is not affected by the *cdc33-1* allele suggests that 57S formation is not similar to that of the 48S complex.

### Stoichiometric analysis of closed-loop factors indicates that the 57S complex is derived from translation complexes nearing the end to translation

We subsequently calculated the absolute stoichiometry of closed-loop factors in the 57S complex so as to determine which of the monosomal and polysomal complexes it was most similar to and hence from which it was most likely derived. In these experiments, as a control, the stoichiometric abundances of these factors were also determined for the 39S complex. The 39S complex (as the 48S complex) should contain protein abundances of closed-loop factors most similar to polysomal complexes to which it would be a precursor. To conduct these experiments we used the same methodology that we have used previously to determine the abundance of these factors in the 77S monosomal translating complex and in polysomes during different stages of the translation process^25,31^. In this general approach, one-step purified material (either Flag-PAB1 or eIF4E-Flag) was split and analyzed separately both by AU-FDS to detect the abundance of a specific tagged GFP protein in the different individually detected complexes and by AU-absorption analysis to determine the total material analyzed in the experiment. Such ratios (AU-FDS/AU-absorption) provide information specifically about protein presence (GFP abundance) in discretely sized individual complexes relative to total material analyzed. Using a standard protein present in particular complexes (STM1-GFP and small ribosomal protein RPS4B-GFP for translation complexes)^25^, we have determined the absolute abundance ratio of other proteins relative to this particular standard in specific complexes in ribosomal complexes^25,31,32^. Table 2 summarizes the results for the stoichiometry of closed-loop factors in polysomal material and 77S translating monosomes after Flag-PAB1 and eIF4E-Flag pull downs for cells experiencing steady-state elongation conditions (glucose-grown cells)^25^. As comparison, we provide our determinations for the stoichiometric ratios of the closed-loop factors in the 39S and 57S complexes.

**Table 2:** Stoichiometric analysis of closed-loop factors in the 57S complex.

Protein/RNA	Flag- PAB1 Polysome	Flag-PAB1 mono-some 77S	Flag-PAB1 57S	Flag-PAB1 39S	eIF4E-Flag polysome	eIF4E-Flag monosome 77S	eIF4E-Flag 57S	eIF4E-Flag 39S
mRNA	100	100	100	100	100	100	100	100
Ribosomes	240	100	100	100	310	100	100	100
PAB1	100	100	100	100	74 ± 6.5	12 ± 0.69	26 ± 3.7	42 ± 9.1
eIF4E	27 ± 3.0	16 ± 0.67	14 ± 2.9	28 ± 9.5	100	100	100	100
eIF4G1	18 ± 1.1	5.6 ± 0.56	6.4 ± 0.91	13 ± 2.3	75 ± 5.6	19 ± 0.78	71 ± 8.6	88 ± 12
eIF4G2	9.4 ± 1.2	3.9 ± 0.028	3.5 ± 1.4	8.3 ± 3.4	61 ± 15	15 ± 3.4	32 ± 11	29 ± 5.4

Stoichiometric calculations were done as described^25^ and as summarized in the text. All values are given relative to 100 mRNA bound by ribosomes. The number of ribosomes in polysomal material was determined as described^25^. Values represent at least three to seven separate determinations. Yeast contain two eIF4G isomers and data is given for each.

Several important observations can be made from these stoichiometric analyses. First, in eIF4E-Flag identified polysomes (i.e., polysomal material containing eIF4E irrespective if they contain poly(A) tails or not), at least 100% of such complexes contain eIF4G (actually 136%, see ref.^25^. for a discussion about the possibility that more than one eIF4G molecule is actually present in each polysomal structure). About 75% of such polysomes are polyadenylated (containing PAB1), implying that a major portion of polysomal material with eIF4E exists in a canonical closed-loop structure, which is expected of translating polysomal structures. Second, the 77S monosomal translation complex when polyadenylated (using Flag-PAB1 pull downs) was more deficient in the eIF4E/eIF4G closed-loop factors (16% and 9.5%, respectively) than polysomal complexes (27% and 27%, respectively) and when containing eIF4E (eIF4E-Flag pull downs), the 77S complex was more deadenylated (only 12% had PAB1 present) than polysomal complexes (74% had PAB1 present) and had less eIF4G (34% compared to 140% in polysomal complexes). Both of these observations indicate that the 77S monosomal complex is nearing the end to translation, having lost eIF4E/eIF4G and containing mRNA that are substantially deadenylated. These results are in complete agreement with an analysis of mRNA presence in monosomal translating complexes^50^. Third, in Flag-PAB1 purified material (mRNA containing polyadenylated tails), the 39S complex contained closed-loop factor abundances (eIF4E at 28% of ribosomal complexes and eIF4G at 21%) most similar to that of polyadenylated mRNA present in polysomes (eIF4E at 27% and eIF4G at 27%) but not those present in 77S monosomal translation complexes (16% with eIF4E and 9.5% with eIF4G) (Table 2). This is the expected result for a 48S complex that is the precursor to the formation of new polysomal complexes that represents about 95% of translation complexes^38^. In contrast, the abundances of closed-loop factors in the 57S complex (14% containing eIF4E and 9.9% containing eIF4G) most resemble the abundances found in polyadenylated 77S monosomal translation complexes (16% containing eIF4E and 9.5% containing eIF4G). As these 77S monosomal complexes are presumed to be nearing the end of functional translation^25^, the 57S complex appears to be derived from translation complexes about to terminate, consistent with 57S complexes forming upon ribosomal dissociation.

Fourth, for 39S complexes identified with eIF4E-Flag, 42% were polyadenylated and eIF4G abundance was 117% of eIF4E. Again, this 39S pool of complexes looks most similar to that of the eIF4E-containing polysomal complexes (74% polyadenylated and 140% with eIF4G), suggesting that the 39S complex is the precursor complex containing mRNA about to be converted into polysomal complexes. For the 57S complexes containing eIF4E, they were more deadenylated and had less eIF4G than eIF4E-containing polysomal complexes: 26% carried PAB1 and about 100% had eIF4G. Because eIF4E-containing 77S monosomal translation complexes were even more deadenylated (only 12% carried PAB1) and had less eIF4G present (only 34%), this pool of eIF4E-containing 57S complexes appear to be derived from both 77S monosomal translation complexes and from polysomal complexes.

We subsequently determined the stoichiometry of the closed-loop factors in the 57S complex following translational repression when termination processes were augmented. As observed under elongation conditions, the 57S complex purified by Flag-PAB1 following the shift of cells to 46 °C for 1 min again displayed an eIF4E (21%) and eIF4G (6.8%) composition most similar to that of the monosomal translation complex (Table 3) in which the abundance of eIF4G was much less than that of eIF4E (16% and 9.5%, respectively) while that of polysomal complexes the eIF4E and eIF4G were identical (27%). As a control, the 39S complex once again was more similar to that of polysomal complexes with nearly equivalent levels of eIF4E and eIF4G (each 20%). These results are similar to those obtained under steady-state elongation conditions.

**Table 3:** Stocihiometric analysis of translation factors in 57S complex under different translation conditions following PAB1-Flag pull downs.

Factor	Elongation	Termination	Initiation	Elongation	Termination	Initiation
	57S Glc+	57S 30 °C to 46 °C 1 min	57S Glu + − 10 min +1 min	39S Glc+	39S 30 °C to 46 °C 1 min	39S Glu + − 10 min + 1 min
eIF4E	14 ± 2.9	21 ± 9.4	5.1 ± 2.1	28 ± 9.5	20 ± 2.5	14 ± 3.8
eIF4G1	6.4 ± 0.91	3.0 ± 2.2	5.3 ± 1.1	13 ± 2.3	7.9 ± 1.7	8.8 ± 1.6
eIF4G2	3.5 ± 1.4	3.8 ± 2.3	3.2 ± 0.45	8.3 ± 3.4	12 ± 8.1	5.3 ± 2.7

Stoichiometric analyses were conducted under the conditions indicated as described in Table 2.

It should be noted that under re-initiation conditions, the 57S complex, while still deficient in eIF4E, now had comparable or greater levels of eIF4G present (8.5%) than eIF4E (5.1%). This result suggests that the 57S may be rebinding eIF4G upon re-initiation and recycling its closed-loop factors back into translation.

### The 57S complex does not contain factors involved in ribosomal dissociation or translation termination

In that the 57S increases in abundance under conditions that mimic translational termination, it is possible several of these factors and those involved in ribosomal dissociation might be present in the 57S complex. Several proteins (HBS1, DOM34, RLI1, and eEF3) play different roles in aiding 80S ribosomal dissociation into its constituent subunits^1,12,14,15^. Using GFP fusions to each of these proteins and following eIF4-Flag purification of translational complexes, we found no HBS1, DOM34, or RLI1 in 80S/77S, 57S, or 39S complexes (Suppl. Figure 3A). Similarly, using Flag-PAB1 pull downs, the translation elongation factor eEF3 was not present in 39S and 57S complexes (Suppl. Figure 3B). Finally, the eRF1 and eRF3 proteins important to translation termination were not present in the 57S or 39S complexes (Suppl. Figure 3C,D) (also ref.^34^). These eRF1 results are in agreement with our previous analyses in which a 57S eRF1-containing complex was devoid of closed-loop factors^31^. These analyses suggest that the 57S complex is not a transitional complex involved in monosomal dissociation or termination.

We also examined whether we could detect defects in the closed-loop interactions that would interfere with the 57S complex formation. Our above analysis with the *cdc33-1* allele indicated that the complex still formed even under conditions when eIF4E was defective in binding the mRNA. Two other types of closed-loop interactions were examined. In the first case, we examined the effect of deleting the N-terminal 300 amino acids of eIF4G1 on the 57S complex. The *ΔN300-TIF4631* allele has been shown to result in an eIF4G1 protein both defective in binding mRNA and PAB1^51^. However, as shown in Suppl. Figure 2E, deleting the N-terminal 300 amino acids of eIF4G1 had no effect on 57S complex formation. Similarly, deleting either the RRM1 domain of PAB1 or the RRM2 domain of PAB1 (either deletion results in a defect in PAB1 binding eIF4G)^52^ had no effect on 57S complex formation (Suppl. Figure 3E,F). These results suggest that closed-loop formation interactions are not critical to formation of the 57S complex.

## Discussion

**Figure 4:**
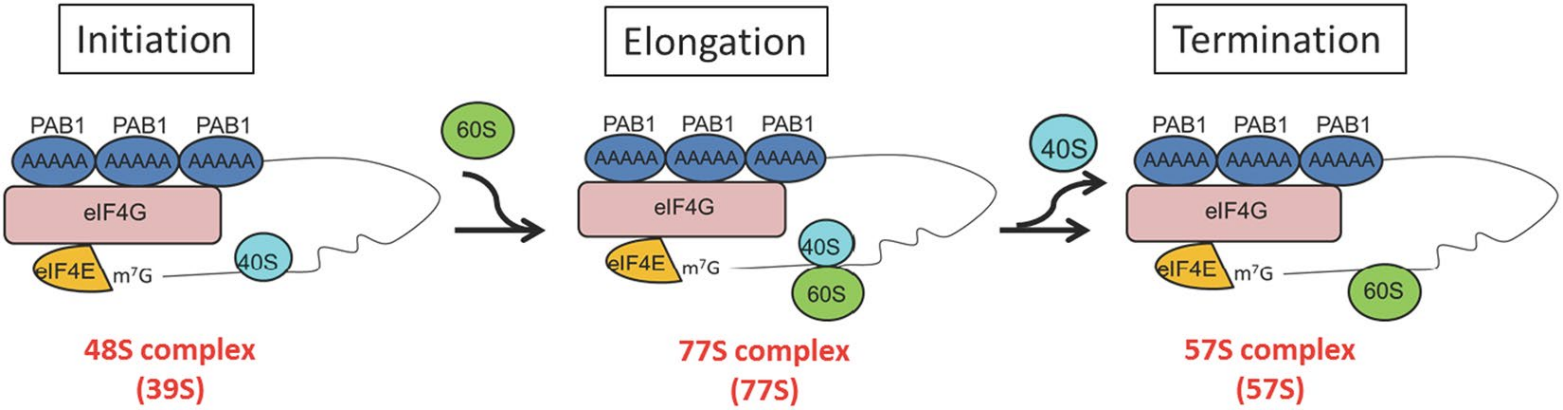
Model for role of 57S complex in translation. A summary is presented of the composition and probable roles of the principal three complexes discussed (39S, 57S, and 77S). The text contains additional models and compositions of these complexes.

We have used different affinity purification steps combined with AU-FDS to characterize translational complexes from the yeast *Saccharomyces cerevisiae*^8,25,31,33,34^. As presented herein, in the course of these studies we have identified a 57S translation complex. The 57S complex contains the 60S ribosome bound to mRNA and the closed-loop factors, eIF4E, eIF4G, and PAB1 (see Fig. 4). Components of the 40S ribosome were not identified in the 57S complex, and other translation initiation factors (eIF2, eIF3, eIF4A, and eIF5) were not present in the 57S complex. eIF3 was not present in the 57S complex even after formaldehyde cross-linking prior to cell lysis in order to maintain its presence in unstable complexes^21,34^. Moreover, the 57S complex was not the product of breakdown of translation complexes after cell lysis since our experiments were conducted in the presence of cycloheximide that maintain such complexes on the mRNA^25,31^. The 57S complex represents, therefore, a novel complex.

Other researchers using sucrose gradient analyses have also identified a 50S-60S complex. For example, Castelli *et al*. 2011, ref.^35^, followed PAB1 and eIF4G presence after crude extracts had been separated by sucrose gradient centrifugation in order to determine which translational complexes they were a part of. Under steady-state glucose growth conditions PAB1 and eIF4G were present in polysomal and monosomal fractions as well as 60S and 40S fractions (Fig. 2D of ref.^35^). Following repression of translation by removal of glucose (when we observe a specific 1.9-fold increased abundance of the 57S complex relative to steady-state glucose conditions) (Table 1 and Fig. 1), Castelli *et al*. 2011 observed that both eIF4G and PAB1 were present in a discrete complex migrating at 60S^35^. Under glucose-growth conditions, this complex could not be clearly identified. Similarly, Hu *et al*. 2009, in their analysis of where polyadenylated and deadenylated mRNA migrated in a similar sucrose gradient analysis of crude extracts, found under glucose depletion conditions (in this case cells were grown on galactose, a non-glucose repressing carbon source) that a discretely visual 60S complex appeared containing both types of mRNA while they were unable to observe this complex under glucose growth conditions (Fig. 2f compared to 2e of ref.^38^). Similar results were obtained by other labs (Fig. 2C of Coller and Parker 2005, ref.^36^. and Figure 5C of Inada *et al*. 2002, ref.^39^). In the Inada *et al*. 2002 paper, it can be noted that the 60S complex that was observed increased in abundance for particular mRNA whose translational initiation had been specifically slowed^39^, implying a role for the 60S complex in translation initiation or a recycling of 60S complexes containing mRNA back into translation, a phenomenon that was consonant with our re-initiation analyses (Tables 1 and 3). These results indicate that the 57S complex has been detected by multiple individuals using different techniques.

The abundance of the 57S complex was shown to increase upon translation termination conditions, supporting the model that upon dissociation of the translating ribosome, some mRNA stays associated with the 60S ribosome. The mRNA that stays associated, if it were to contain eIF4E, also contains only some PAB1 and eIF4G, and if it were to contain PAB1 contains only some eIF4E and eIF4G. In general, because such terminating ribosomes have lost substantial amounts of these closed-loop factors, only a small percentage of these 57S complexes would be expected to be in a closed-loop complex, and that is what we observe. A number of factors involved in ribosomal dissociation were found not to be present in the 57S complex (RLI1, DOM34, HBS1, eEF3, eRF1, and eRF3), implying that the 57S complex is not a transient intermediate in this process but is rather a final product that appears after dissociation of the translating ribosome has taken place.

Although we have identified a 57S complex, its function is not clear. For example, we were not able to show that disrupting eIF4E-mRNA, eIF4G-PAB1, or eIF4G-mRNA interactions had any effect on the formation of the 57S complex that could be tied to specific translation defects. It is, therefore, possible that rather than the 57S complex forming specifically upon translation termination, the 57S complex exists as a sort of depot for closed-loop factors and the 60S ribosome subunit. In this model, the 57S complex would maintain these components at a high concentration so as to avoid ribosomal or mRNA surveillance pathways. Other types of experiments would be necessary to establish how the mRNA binds the 60S ribosome and whether the mRNA of this complex and its closed-loop factors do indeed recycle into translation.

In addition to the 57S complex, we also identified a 39S complex that contained 40S ribosomal components, mRNA, and closed-loop factors. We also found that eIF3 was not stably present in the 39S complex unless formaldehyde cross-linking was conducted prior to lysis of the cells. The components of this 39S complex make it most similar to the 48S pre-initiation complex. In particular, formaldehyde is known to stabilize the 48S complex and allow retention of initiation factors such as eIF3^21,34^. In regards to the identification of the 48S complex migrating at 39S, previous analysis of the size of the 48S complex by sucrose gradient analysis actually does tend to position it slightly under or at 40S and not at 48S (Fig. 3D of ref.^45^). This result is consistent with our AUC analysis (which would be used to define the real S value). Based on the presumed asymmetry of a ribosomal complex bound to mRNA, the 48S ribosomal complex would be expected to migrate slower following AUC analysis than its actual size^8^. The definition of the actual S values for 43S and 48S complexes was not based on AUC analysis but rather on the expected increase in molecular weight by adding initiation factors and/or mRNA to a 40S ribosomal subunit that had been subjected to AUC analysis in yeast^37^. An asymmetric 48S complex might be expected to migrate as low as 36S if it had a 2:1 length to width ratio. Similarly, the translating monosomal complex, if spherical, should have an S value of 84S based on its mass (on average 4.3 MDa for a normal mRNA of about 0.5 MDa)^53^, but it migrates at 77S due to its presumed asymmetry^8^.

It should be noted that the 39S complex increased in abundance following one of the two conditions that mimicked termination. Some of the 39S complex that we observe may be that of the known 40S subunit association with the mRNA that takes place upon ribosomal stalling^40,41^ and may not be the same as the 48S complex. Relatedly, the 20S complex that we observe that contains both PAB1 and eIF4E lacks ribosomes. In that this 20S complex lacked eIF4E and eIF4G with Flag-PAB1 pull downs and lacked PAB1 and eIF4G with eIF4E-Flag pull downs, it is likely that this 20S complex, presumably containing mRNA due to the presence of eIF4E and PAB1, probably consists of two separate pools of such mRNA that are mutually exclusive. Also, neither of the two mRNA we analyzed (*MFA2* or *PGK1*) appeared present in this 20S complex, implying that only a subset of mRNA are actually in these 20S complexes. Clearly, additional techniques will be required to clarify both the identities of these 39S and 20S complexes and functional roles in translational function.

## Materials and Methods

### Yeast strains and growth conditions

Yeast strains carrying GFP fusions to particular translational factors have been previously described^8,30^. All strains carrying GFP fusions except for that containing eIF4G2-GFP were isogenic with the genotype *Matα ura3 his3 leu2 met15*, with the GFP fusion marked with the *HIS3* gene^8^. The strain carrying eIF4G2-GFP was RP2384, *Mata leu2-3*,*112*, *trp1 ura3-52 his4-539 cup1::LEU2/PGK1pG/MFA2pG TIF4632-GFP::G418*. Previous studies have shown no difference in AU analysis for strains isogenic to RP2384 and to the other strains used^8^. All strains were transformed with either of two plasmids as indicated in the text: YC776 (*Flag-PAB1 URA3*) or YC801 (*eIF4E-Flag URA3*). Cells were grown at 30 °C to mid-log phase in synthetic complete medium with appropriate amino acids as described before^52^. Generally, 200 mL of cells were used for AU-FDS analyses. Cell lysis and Flag pull downs have been described^8^. For glucose depletion, cell pellets from the undepleted medium were washed and then resuspended in fresh medium lacking glucose for 10 min. Glucose re-addition experiments were conducted by adding the requisite amount of glucose (2%) directly to glucose depleted cultures for one minute. Translation termination conditions upon the stress of heat shock was monitored following shifting of cells to 46 °C for 1 min^8,46^. Cycloheximide was added to growing cultures at a concentration of 100 μg/mL as described^8^.

### AU analyses

Flag eluted samples (350 µL) were subjected to AU analysis by using A_230_ absorption (in some cases A_260_ was also conducted) or a fluorescence detection system (AU-FDS)^28,29^ to detect GFP-fusion proteins^8,31–34,54^. Previous analyses, including western analyses and extensive mass spectrometric analyses, have shown that in control immunoprecipitations of extracts obtained from strains lacking a Flag-tagged protein, closed-loop factors such as PAB1, eIF4E, and eIF4G are in very low abundances^52^. Also, mass spectrometric analyses on RNase treated samples from strains containing Flag-PAB1 indicate that these closed-loop factors are no longer immunoprecipitated^52^. Previous AUC analyses have also shown that immunoprecipitations conducted in strains lacking Flag-PAB1are deficient in translation complexes^8^.

All analytical ultracentrifugation experiments were conducted at 20 °C and at a rotor speed of 15,000 rpm. At least 150 scans for AU-FDS experiments were obtained and at least 75 scans for AU-A_230_ analysis. Data were analyzed by SEDFIT software (version 1501b) as described previously^8^. Stoichiometric analyses were done on samples that were split and run at the same time on two different AUC instruments, one for AU-FDS analysis and one for AU-A_230_ analysis.

## Electronic supplementary material

Supplementary Information
