# Supplementary material for: Identification of a 57S translation complex containing closed-loop factors and the 60S ribosome subunit
Source: Sci Rep. 2018 Jul 31;8:11468. doi: 10.1038/s41598-018-29832-6 (PMC6068138; doi:10.1038/s41598-018-29832-6)

Supplementary Information

Identification of a 57S translation complex containing closed-loop factors and the 60S ribosome subunit

Clyde L. Denis, Thomas M. Laue, and Xin Wang

Supplementary Table 1. Relative levels of proteins in the 77S monosomal translating complex during different stages of translation.

	Flag-PAB1			
Factor	Initiation<br>Glu + - 10 min +1 min	Elongation<br>Glu +	Termination<br>$30^{o}C$ to $46^{o}C$ 1 min	Termination<br>Glu + - 10 min
mRNA	100	100	100	100
80S ribosome	100	100	100	100
PAB1	100	100	100	100
eIF4E	23 ± 1.7	16 ± 0.67	16 ± 0.83	13 ± 2.1
eIF4G1	8.7 ± 1.0	5.6 ± 0.56	6.7 ± 0.36	3.9 ± 0.28
eIF4G2	6.2 ± 0.44	3.9 ± 0.028	4.7 ± 0.47	4.8 ± 2.2
eRF1	0.97 ± 0.069	0.97 ± 0.083	2.1 ± 0.18	3.8 ± 0.26

Legend: Stoichiometric analyses were conducted as described in Table 1 to 3 under the conditions indicated.

Supplementary Figure 1. AU-FDS analyses of ribosomal protein presence in the 39S and 57S complexes following Flag-PAB1 pull downs. AU-FDS analyses were conducted as described in Figure 1 using the GFP tagged proteins as Indicated.

Supplementary Figure 2. AUC analyses of translation factors. AUC analyses were conducted as described in Figures 1 and 2.

A. AU-FDS analyses on translation initiation factors as indicated.

B. Same as A except formaldehyde was added as indicated.

C. Same as Figure 3A except following eIF4E-Flag pull downs.

D. Crude extracts from isogenic strains carrying either wild-type *CDC33* or mutated *cdc33-1* were subjected to Flag-PAB1 pull downs at 37°C.

E. Same as D., except strain EPY88 (*tif4632::ura3*) (wild-type but lacking the eIF4G2 protein) carrying either the wild-type *TIF4631* or the deleted allele (*ΔN300-TIF4631*).[51]

Supplementary Figure 3. AU-FDS analyses of ribosomal dissociation and translation termination factors.

A-D. Experiments were conducted as described in Figure 1 and 2 with the GFP tagged factors as indicated.

E. Same as above, except strain AS319/YC505 (*Flag-PAB1-ΔRRM1*) was used.[52]

F. Same as E. above, except strain AS319/YC506 (*Flag-PAB1-ΔRRM2*) was used.[52]

Supplementary Figure 1A

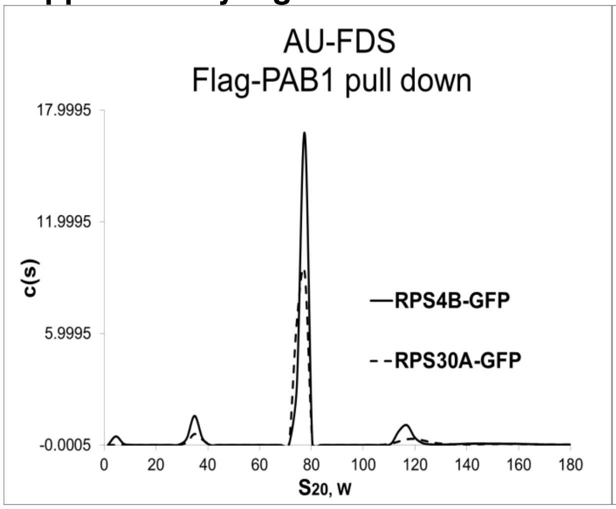

1B

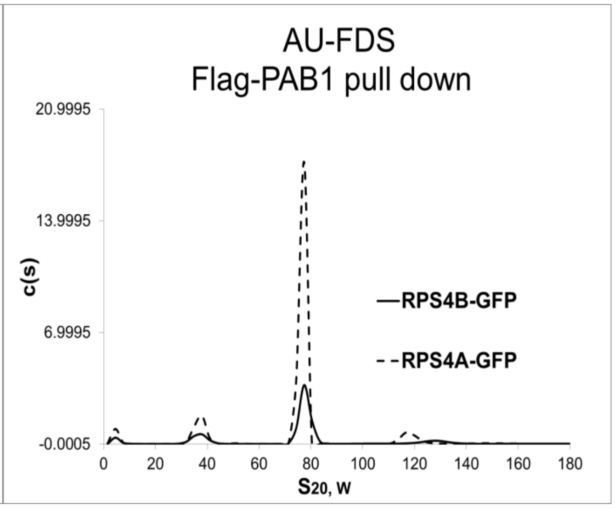

1C

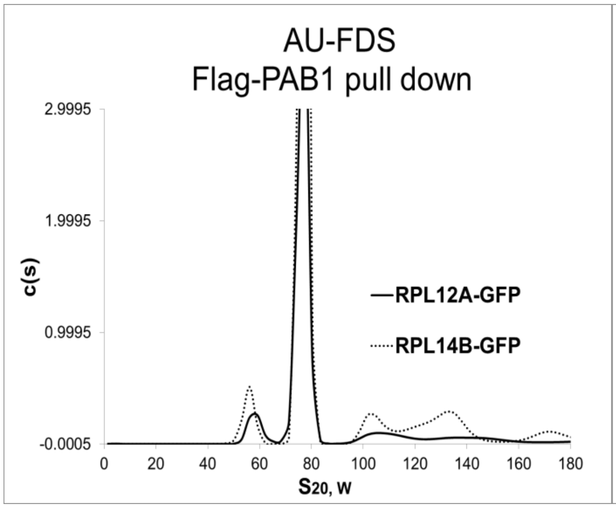

1D

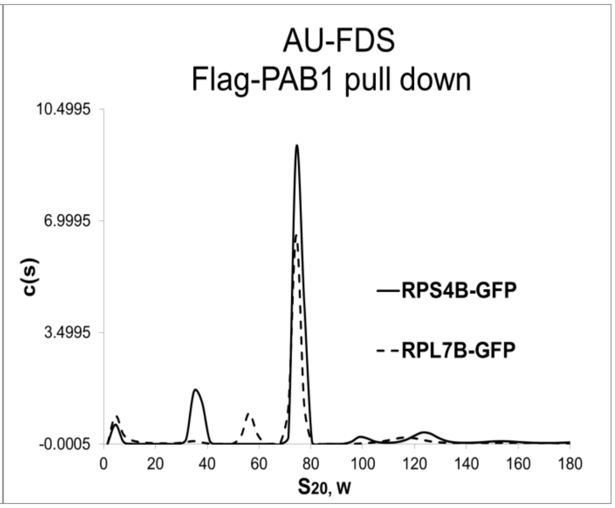

1E

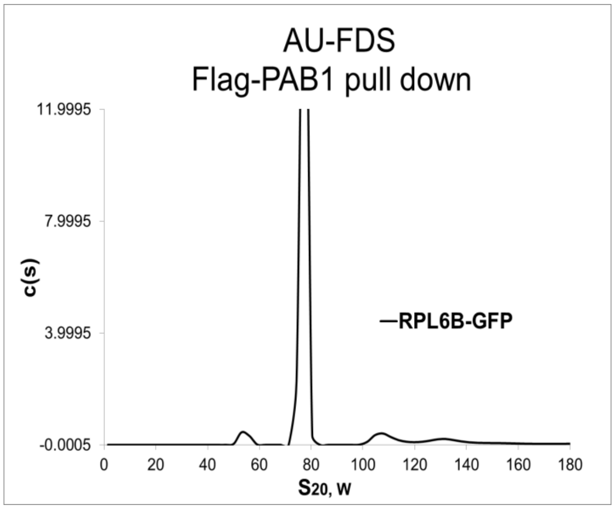

Supplementary Figure 2A

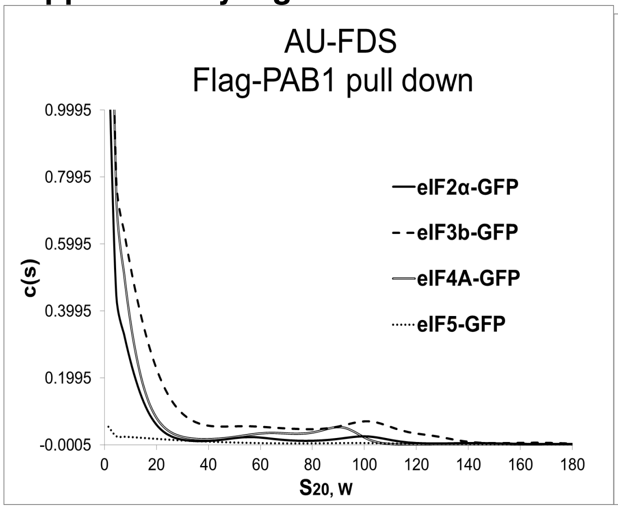

2B

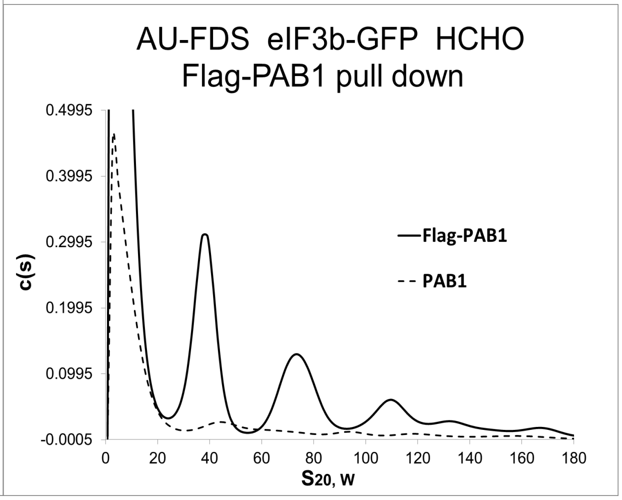

2C

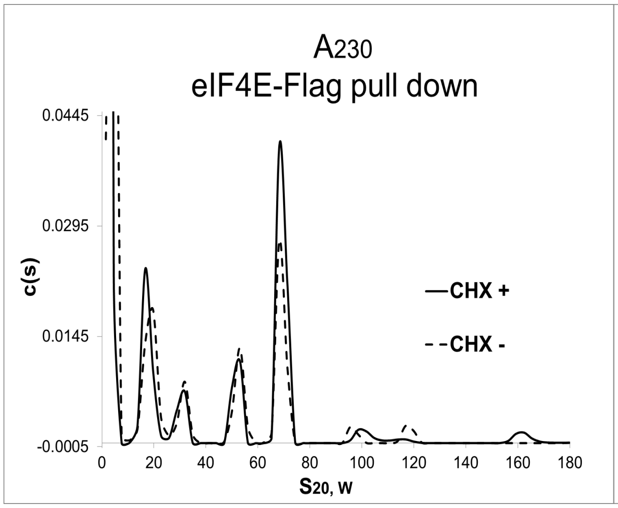

2D

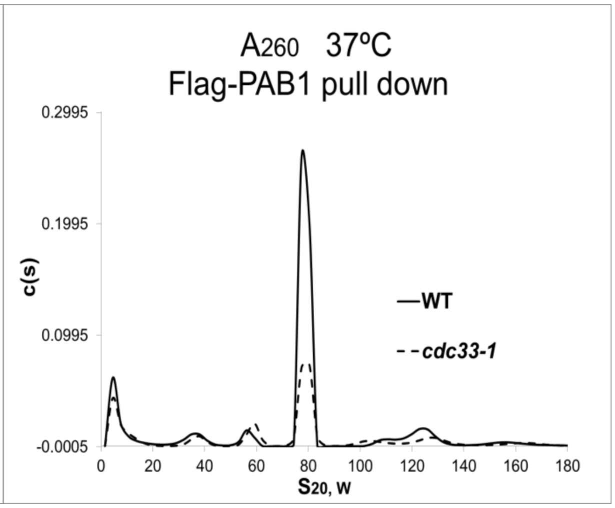

2E

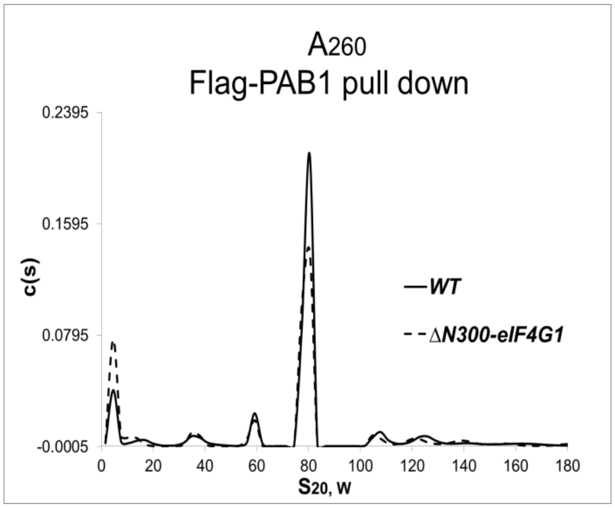

Supplementary Figure 3A

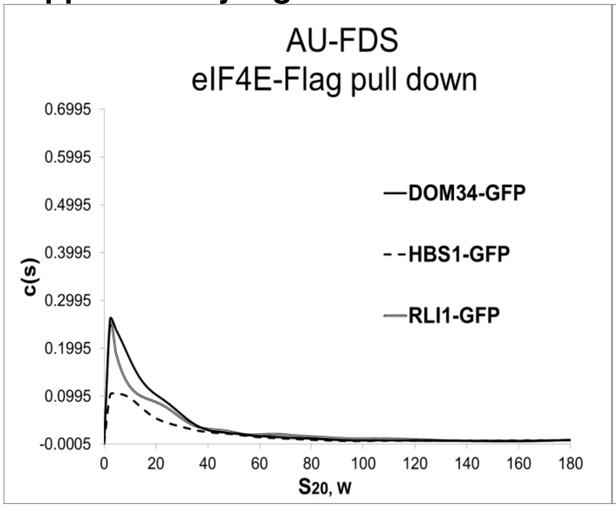

3B

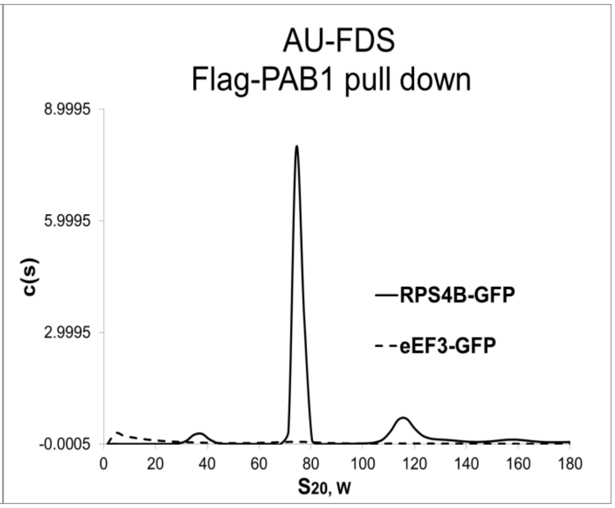

3C

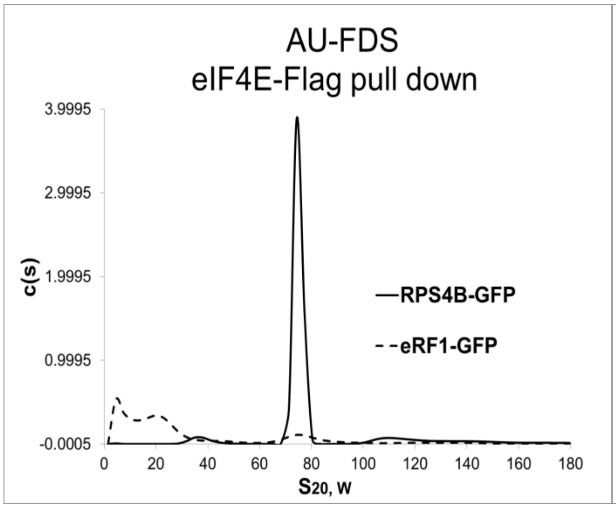

3D

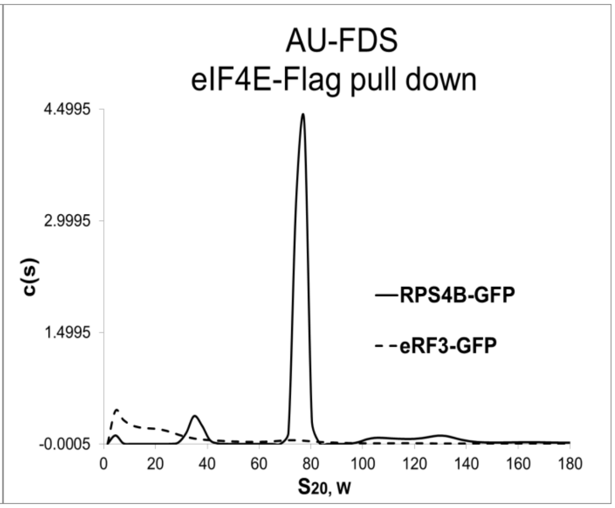

3E

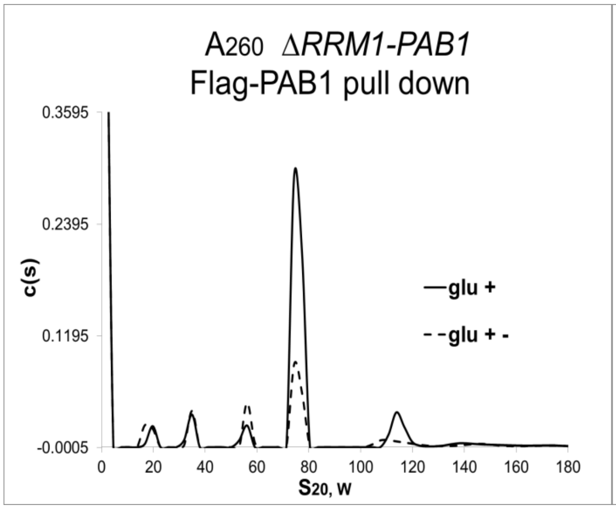

3F

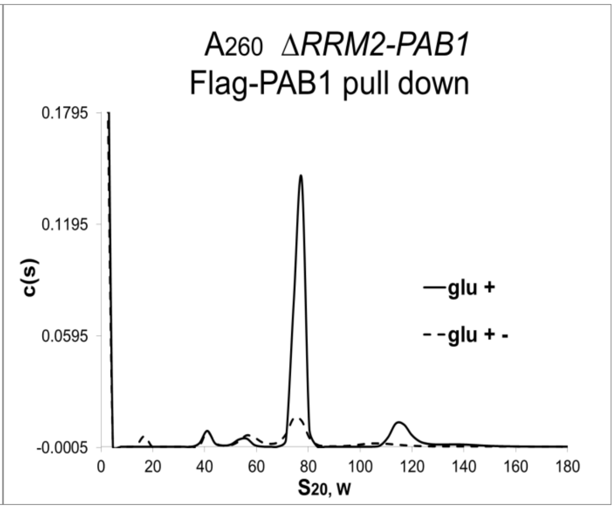

Supplement: Supplementary file 1 — Supplementary Information [file 41598_2018_29832_MOESM1_ESM.pdf]
